# Knowledge of breast cancer, willingness and barriers to mammography screening among rural women in Enugu State, Nigeria

**DOI:** 10.4314/ahs.v23i3.34

**Published:** 2023-09

**Authors:** Lawreta Ijeoma Abugu, Evelyn Nwanebe Nwagu, Adaustin Ifeoma Okeke, Amelia Ngozi Odo

**Affiliations:** Department of Human Kinetics and Health Education, University of Nigeria, Nsukka

**Keywords:** Mammography, breast cancer, screening practice, knowledge

## Abstract

**Background:**

Breast cancer is a serious public health threat. Mammography is the most reliable screening method that detects breast cancer early, enabling early onset of treatment which improves the prognosis of the disease.

**Objectives:**

To determine women's knowledge of breast cancer, as well as barriers and willingness of women to participate in mammography screening.

**Methods:**

Using the cross-sectional survey design, we sampled and studied two rural communities of Enugu State, Nigeria. Two researcher-made questionnaires were used for the study. Frequencies, percentages, chi-square and regression analysis were employed in data analysis.

**Results:**

Only 11.4 percent of study participants had good knowledge of breast cancer. There were significant differences in knowledge of breast cancer based on level of education (χ^2^ = 15.670; p =.001), monthly income (χ^2^ =6.954; p = .021) and ever screened (χ^2^ =5.242; p =.015). Lack of money (48.0%) and lack of knowledge (30.4%) were the most reported barriers to breast cancer screening. Women that had ever screened were 92.3% less likely willing to be screened than those never screened (aOR: .077, 95%CI .011-.522, p=.009).

**Conclusion:**

Health Education should be combined with improving women's economic status and subsidizing the cost of screening to increase breast cancer screening practice.

## Introduction

Breast cancer is a serious public health threat especially in developing countries. The disease has been identified as the most frequently occurring cancer in Nigeria and the second leading cause of death in women world-wide^1^. In developed countries breast cancer screening and early presentation has changed the dismal outcome of the disease, where despite increasing incidence, the morbidity and mortality are declining^2^. However, in developing countries especially Nigeria, there is an alarming increase in incidence of breast cancer. Nigeria with a population of 200 million persons had breast cancer incidence rate of 22.7% and mortality of 16.4% in 2018^3^. In a tertiary institution in Nigeria, malignant breast lesions increased from 26.6% in 2006 to 67.5 % in 2013^4^. Breast cancer has better prognosis when detected and managed early. Education to increase knowledge; and screening to promote early diagnosis are the two major components of early detection of breast cancer^5^.

Unfortunately, most breast cancer presentations in Nigeria and other African countries are at advanced stage of the illness. More than 70% of breast cancer patients present with locally advanced or metastatic breast cancer_6_. Inattention to breast cancer screening among other factors has been advanced as the reason for late presentation of patients with breast cancer^6^, ^7^, ^8^.

Although the Nigerian National Cancer Control Plan (NNCCP) 2018-2022 has one of its goals (goal 2) to make screening services and early detection of cancer available for all Nigerians^9^, this cannot be achieved when women especially those in rural location where greater percentage reside are not aware of the screening services. Furthermore, goal five of the NNCCP 2018-2022 is to increase cancer awareness and advocate for cancer control amongst population with one of the objectives to conduct effective cancer awareness and sensitization activities across the 36 states and FCT. Thus, this study is poised to contribute meaningfully in achieving these goals.

Mammography is the most reliable screening method for breast cancer as it picks tumour very early enough before it can be noticed by the individual^15^. The American Cancer Society recommendation is that women aged 45 to 54 years old have a yearly mammogram while those aged 55 and above should have it every two years^10^. Currently, Nigeria has no National breast cancer screening guideline, therefore, screening recommendations are based on international guidelines^11^. Screening for breast cancer has been shown to improve prognosis of the disease. However, rural women may not be able to partake in this preventive health exercise for several reasons. The reasons could range from lack of awareness about breast cancer screening and its benefits to poverty and no means of income to undertake the screening activity. Studies have shown that there is generally low utilization of mammography among all groups of eligible women especially low income and elderly women^12^.

The cost of mammography coupled with lack of knowledge may prevent women from having the test. The cost of mammography in Nigeria ranges from 12,000 naira in public hospitals to 25, 000 or more in private laboratories^13^. Most rural women who do not have a steady income may not be able to undertake this preventive health action.

This study specifically determined: women's knowledge of breast cancer; their willingness and barriers to participate in mammography screening; and factors associated with knowledge and willingness to screen for breast cancer.

## Methods

### Study design and area

We adopted cross sectional survey and carried out the study in Enugu state, South East Nigeria. The State is made up of 17 Local Government Areas divided into three senatorial zones for administrative purposes. Two of the three senatorial zones that are predominantly rural were used for the study. The citizens are mainly Igbos and belong mostly to the Christian religion. Women in this part of the country are mostly petty traders and artisans and may not be exposed to breast cancer knowledge and screening opportunities.

### Population and sample

Population consisted of all women of reproductive age in Enugu State. Two of the three senatorial zones were purposively selected because they are predominantly rural. Two communities (one from each of the sampled senatorial zones) of Enugu state were selected using simple random sampling technique. We carried out the study in two orthodox churches (Anglican Church and the Roman Catholic Church) in each of the rural communities. Women in these churches have their annual convention during August therefore, we utilized the opportunity to meet with the women. Informed consent was obtained from each participant. Participation was voluntary and only those that met the inclusion criteria were recruited and used for the study.

Inclusion criteria were those aged 15 years and above, and willing to partake in the study. Exclusion criteria were those that were on breast cancer treatment. A total of 276 women were approached in the four churches where the study was carried out, 188 gave verbal consent to participate in the study. Of these, three were excluded using the exclusion criteria. Therefore, 185 women participated in the study (98 & 87) from each of the communities. However, only 158 correctly filled out their questionnaires and therefore, were used for data analysis representing 85% return rate.

### Study instrument

Two questionnaires developed by the researchers were used for the study. The first questionnaire titled “Knowledge of Breast Cancer among Rural Women Questionnaire (KBCQ)” sought information on knowledge about breast cancer. This instrument had two sections. Section A elicited information on participant socio-demographic and clinical characteristics, while section B elicited information on participants' knowledge of breast cancer. Questions were asked on five dimensions of knowledge of breast cancer thus concept — five questions, risk factors — fourteen questions, signs and symptoms — eight questions, prevention —four questions and screening -five questions. Each of these dimensions had multiple answers and participants were asked to tick as many as they know of. The second questionnaire titled “Barriers to and Willingness to Participate in Mammography Screening Questionnaire (BWPMSQ)” was used to elicit information on participant's barriers to and willingness to participate in mammography screening. Participants' names were not included in the instruments. The instruments were validated by three research experts. Reliability of the instruments were determined using Cronbach alpha statistic with a reliability index of .82 and .70 for KBCQ and BWPMSQ respectively.

### Data collection

We collected data during the annual general meeting of the women in the churches. On the first day of data collection, the researchers together with research assistants visited the Roman Catholic Churches and administered the KBCQ and the BWPMSQ to the participants. The second day, the researchers together with research assistants visited the Anglican Churches and also administered the KBCQ and the BWPMSQ to consenting participants. The instruments were collected back immediately on the spot. Data were collected on 27^th^ and 28^st^ August, 2021.

### Data analysis

We used SPSS version 21 to analyse the data collected from the questionnaires. One hundred and fifty-eight correctly filled questionnaires were used for analysis. For questions on knowledge of breast cancer, each correct response was scored one point while incorrect response was scored zero. The scores for each subsection and the total score were calculated. Bloom's cut off point was used to determine good or poor knowledge of breast cancer whereby a score of 60% and above was regarded as good knowledge whereas below 60% was regarded as poor knowledge 14.

However, the authors modified the criteria to 50% cut off due to the rural nature of the study area. Frequencies and percentages were used to find proportion of the women that had good and poor knowledge of different dimensions of breast cancer and its screening while chi square statistics was used to find association between outcome variables and participants' characteristics. Frequencies and percentages were also used to find barriers to screening and willingness to screen for breast cancer among the women. For factors associated with knowledge of breast cancer and willingness to participate in mammography, we performed logistic regression analysis to determine predictors of the outcome variables. Only variables with a *p* value less than .05 in the univariable analysis were included in the multivariable model. Multicollinearity was checked by the Variance Inflation Factor (VIF). A benchmark IVF less than 10 was used to indicate absence of collinearity before including the variable in the multivariable analysis^15^. The fitness of the model was assessed using Hosmer-Lemeshow test while goodness of fit was assessed by Nagelkerke R^2^

### Ethical consideration

Ethical Approval for this study was obtained from Enugu State Ministry of Health (MH/MSD/REC21/228). Informed consents were obtained verbally from the mothers and only those who consented were involved in the study.

## Results

The socio-demographic characteristics of participants in Table 1 shows that almost one half of the women (44.9%) were aged 55 years and above with the mean age of 50.96, ± 12.99. More than half had primary education as their highest level of education (53.2%) and were married (59.5%). Majority had four or more children (82.9) while more than two-thirds of the women were self-employed (79.1). Most women earn less than 50,000 naira as monthly income (89.9%).

**Table 1:** Socio-demographic and clinical characteristics (n=158)

Characteristics	Frequency	Percentage (%)
**Age range (years)**		
15 – 39	27	17.1
40-55	60	38.0
56-70	71	44.9
**Highest level of education**		
No formal education	34	21.5
Primary education	84	53.2
Secondary education	25	15.8
Tertiary education	15	9.5
**Marital status**		
Married	94	59.5
Widowed	58	36.7
Divorced/separated	6	3.8
**Parity**		
None	7	4.4
1-3	20	12.7
4 and above	131	82.9
**Occupation**		
Civil servant	23	14.6
Self employed	125	79.1
Unemployed	10	6.3
**Monthly income (Naira)**		
Less than 50,000	142	89.9
50,000 and above	16	10.1
**Ever diagnosed of breast cancer**		
No	153	96.8
Yes	5	3.2
**Family history of breast cancer**		
No	151	95.6
Yes	7	4.4
**Ever done any breast cancer screening**		
No	113	71.5
Yes	45	28.5
**Screening method ever done**		
None	113	71.5
Breast self-examination	25	15.8
Clinical breast examination	14	8.9
Mammogram	6	3.8

On the clinical characteristics, most respondents had never been diagnosed of breast cancer (96.8%), and did not report family history of breast cancer (95.6%). More than two-thirds (71.5%) of the respondents had never done any breast cancer screening while only 3.8% had ever done mammography screening Finding from Table 2 shows that most women (88.6%) had poor knowledge of breast cancer.

**Table 2:** Knowledge of women on Breast cancer

Knowledge categories	Poor knowledge	Good knowledge
	f (%)	f (%)
Concept of breast cancer	131 (82.9)	27 (17.1)
Risk factors	149 (94.3)	9 (5.7)
Signs and symptoms	139 (88.0)	19 (12.0)
Prevention	147 (80.4)	31 (19.6)
Screening	131 (82.9)	27 (17.1)
**Overall knowledge**	**140(88.6)**	**18 (11.4)**

Table 3 shows that only level of education (χ^2^ = 15.670; *p*=.001), monthly income (χ^2^ =6.954; *p*= .021) and ever screened (χ^2^ =5.242; *p*=.015) were significantly associated with knowledge of breast cancer. Table 4 shows that ever participated in breast cancer screening (χ^2^ =4.788; *p*=.043) and screening method ever (χ^2^ =8.030; *p*=.045) were significantly associated with willingness to screen for breast cancer. The most reported barrier to breast cancer screening as shown in Table 5, was lack of money (48.0%) and lack of knowledge of what the condition is all about (30.4%).

**Table 3:** Knowledge of breast cancer with socio-demographic and clinical characteristics

	Knowledge Categories							
	Concept	Risk factor	Signs &symptoms	Prevention	Screening	Overall knowledge	χ^2^	P
Characteristics	F (%)	F (%)	F (%)	F (%)	F (%)	F (%)		
**Age range (years)**								
Below 40	7(25.9)	1(3.7)	4(14.8)	6(22.2)	3(11.1)	2(7.4)		
40-55	8(13.3)	4(6.7)	7(11.7)	9(15.0)	10(16.7)	6(10.0		
Above 55	12(16.9)	4(5.6)	8(11.3)	16(22.5	14(19.7)	10(14.1)	1.050	.529
**Level of education**								
No formal	3(8.8)	2(5.9)	4(11.8)	7(20.6)	6(17.6)	4(11.8)		
education								
Primary education	13(15.5)	4(4.8)	8(9.5)	15(17.9)	15(17.9)	8(9.5)		
Secondary	3(12.0)	0(0.0)	1(4.0)	4(16.0)	1(4.0)	0(0.0)		
education								
Tertiary education	8(53.3)	3(20.0)	6(40.0)	5(33.3)	5(38.3)	6(40.0)	15.670	.001*
**Marital status**								
Married	22(23.4)	8(8.5)	14(14.9)	19(20.2)	17(18.1)	12(12.8)		
Widowed	4(6.9)	0(0.0)	4(6.9)	10(17.2)	9(15.5)	5(8.6)		
Separated/divorced	1(16.7)	1(16.7)	1(16.70	2(33.3)	1(16.7)	1(16.7)	.782	.676
**Parity**								
None	2(28.6)	1(14.3)	2(28.6)	1(14.3)	2(28.6)	1(14.3)		
1-3	1(5.0)	0(0.0)	1(5.0)	4(20.0)	4(20.0)	1(5.0)		
4 and above	24(18.3)	8(6.1)	16(12.2)	26(19.8)	21(16.0)	16(12.2)	.955	.620
**Occupation**								
Civil servant	6(26.1)	2(8.7)	2(8.7)	5(21.7)	3(13.0)	3(13.0)		
Self employed	17(13.6)	5(4.0)	13(10.4)	21(16.8)	21(16.8)	12(9.6)		
Unemployed	4(40.0)	2(20.0)	4(40.0)	5(50.0)	3(30.0)	3(30.0)	3.890	.143
**Monthly income (Naira)**								
Less than 50,000	22(15.5)	5(3.5)	16(11.3)	25(17.6)	24(16.9)	13(9.2)		
50,000 and above	5(31.3)	4(25.0)	3(18.8)	6(37.5)	3(18.8)	5(31.3)	6.954	.021*
**Ever diagnosed breast cancer**								
No	27(17.6)	9(5.9)	19(12.4)	31(20.3)	26(17.0)	18(11.8)		
Yes	0(0.0)	0(0.0)	0(0.0)	0(0.0)	1(20.0)	0(0.0)	.664	.542
**Family history of breast cancer**								
No	27(17.9)	9(6.0)	18(11.9)	30(19.9)	26(17.2)	17(11.3)		
Yes	0(0.0)	0(0.0)	1(14.3)	1(14.3)	1(14.3)	1(14.3)	.061	.579
**Ever screened**								
No	19(16.8)	8(7.1)	17(15.0)	29(25.7)	24(21.2)	17(15.0)		
Yes	8(17.8)	1(2,2)	2(4.4)	2(4.4)	3(6.7)	1(2.2)	5.242	.015*
**Screening method ever**								
None	19(16.9)	8(7.1)	17(15.0)	29(25.7)	24(21.2)	17(15.0)		
Breast self exam	4(16.0)	1(4.0)	2(8.0)	2(8.0)	3912.0)	1(4.0)		
Clinical exam	1916.7)	0(0.0)	0(0.0)	0(0.0)	0(0.0)	0(0.0)		
Mammogram	3(21.4)	0(0.0)	0(0.0)	0(0.0)	0(0.0)	0(0.0)	5.418	.144

* Significant

**Table 4:** Willingness to participate in mammography with socio-demographic and clinical characteristics

Characteristic		Not Willing	Willing	χ^2^	p
F (%)	F (%)				
**Age range (years)**					
15-39		2 (7.4)	25 (92.6)		
40-55		4 (6.7)	56 (93.3)	1.357	.503
56-70		2 (2.8)	69 (97.2)		
Total		8 (5.1)	150 (94.9)		
**Level of education**					
No formal education		0 (0.0)	34 (100)		
Primary education		4 (4.8)	80 (95.2)		
Secondary education		3 (12.0)	22 (88.0)	4.412	.220
Tertiary education		1 (6.7)	14 (93.9)		
Total		8 (5.1)	150 (94.9)		
**Marital status**					
Married		5 (5.3)	89 (94.7)		
Widowed		3 (5.2)	55 (94.8)	.334	.846
Separated/divorced		0 (0.0)	6 (100)		
Total		8 (5.1)	150 (94.9)		
**Parity**					
None		0 (0.0)	7 (100)		
1-3		2 (10.0)	18 (90.0)	.451	.484
4 and above		6 (4.6)	125 (95.4)		
Total		8 (5.1)	150 (94.9)		
**Occupation**					
Civil servant		3 (13.0)	20 (87.0)		
Self-employed		5 (4.0)	120 (96.0)	3.874	.144
Unemployed		0 (0.0)	10 (100)		
Total		8 (5.1)	150 (94.9)		
**Monthly income (Naira)**					
Less than 50,000		8 (5.6)	134 (94.4)		
50,000 and above		0 (0.0)	16 (100)	.949	.417
Total		8 (5.1)	150 (94.9)		
**Ever diagnosed of breast cancer**					
No		8 (5.2)	145 (94.8)		
Yes		0 (0.0)	5 (100)	.275	.600
Total		8 (5.1)	150 (94.9)		
**Family history of breast cancer**					
No		8 (5.3)	143 (94.7)		
Yes		0 (0.0)	7 100)	.391	.532
Total		8 (5.1)	150 (94.9)		
**Ever screened for breast cancer**					
No		3 (2.7)	110 (97.3)		
Yes		5 (11.1)	40 (88.9)	4.788	.043*
Total		8 (5.1)	150 (94.9)		
**Screening method ever**					
None		3 (2.7)	110 (97.3)		
Brest self-exam		4 916.0)	21 (84.0)		
Clinical exam		0 (0.0)	6 (100)	8.030	.045*
Mammogram		1 (7.1)	13 (92.9)		
Total		8 (5.1)	150 (94.9)		

* Significant

**Table 5:** Barriers to participation in mammogram

Barriers	No F (%)	Yes f (%)
Lack of knowledge of what it is all about	110(69.6)	48(30.4)
Lack of money	90(57.0)	68(48.0)
Afraid of what result could be	134(84.8)	24(15.1)
No time to go for screening	150(94.9)	8(5.1)
Not necessary for me	148(93.7)	10(6.3)
Lack of knowledge of screening importance	135(85.4)	23(14.6)
Do not think I can ever develop breast cancer	131(82.9)	27(17.1)

Table 6 shows that women who had tertiary education were five times more likely to have breast cancer knowledge than those with no formal education (OR: 5.000, 95% CI 1.152-21.706; p=.032). Those who earned 50,000 naira and above monthly were four and a half times more likely to have knowledge than those who earned less than 50,000 naira monthly (OR: 4.510, 95% CI 1.357-14.995; P=.014). Women who had ever screened were 87.2% less likely to have breast cancer knowledge than those that had never screened (OR: .128, 95% CI .017-.993; P=.049). Thus, women's level of education, monthly income and having ever screened for breast cancer significantly contributed to knowledge of breast cancer and its screening. Participant's age, marital status, parity, occupation, ever diagnosed of breast cancer and family history of breast cancer did not contribute significantly to the model. On willingness to screen, those that had ever screened were 78.2% less likely willing to screen than those never screened (OR:.218, 95% CI .050-.955; P=.043.

**Table 6:** Univariable and multivariable analysis of factors associated with knowledge and willingness to screen for Breast Cancer

Factors	Univariable OR (95%CI)	P-value	Multivariable aOR(95%CI)	P-value
**Knowledge of breast cancer**				
**Level of education**		.036		
No formal education				
Primary education	.789(.221-2.818	.716	.305(.062-1.502)	.114
Secondary education	.000(.000)	.998	.190(.049-.741)	.017*
Tertiary education	5.000(1.152-21.706)	.032	.000(.000)	.998
**Ever screened**		.000		
No^c^				
Yes	.128(.017-.993)	.049	.131(.016-1.076)	.059
**Monthly income(naira)**		.000		
Less than 50,000^b^				
50,000 and above	4.510(1.357-14.993)	.014	.339(.085-1.343)	.123
**Willingness to screen for breast cancer Ever screened**		.000		
No				
Yes	.218(.050-.955)	.043	.077(.011-.522)	.009*

* Significant

Hosmer-Lemeshow χ^2^(5) = 2.944; p = .709

Nagelkerke R^2^ = .265

Variance Inflation Factor (VIF) = 1.053, 1,050 and 1.004 (level of education, monthly income and ever screened) respectively

Ref Groups: Level of education =no formal education; Monthly income= Less than 50,000^b^ ; Ever screened = No CI = Confidence Interval; OR =Odds Ratio; aOR= adjusted Odds Ratio; * = Significant.

In the multivariable analysis, only women's level of education contributed significantly to knowledge of breast cancer (Table 6). Monthly income and having ever screened for breast cancer did not significantly contribute to the model. Women with secondary education were 81% less likely to have knowledge than those with no formal education (a OR: .190, 95%CI .049-.741; p=.017). Women that had ever screened were 92.3% less likely willing to be screened than those never screened (a OR: .077, 95% CI .011-.522, p=.009).

## Discussion

Our study found out that the women generally had poor knowledge of different aspects of breast cancer. Knowledge was worst for risk factors for breast cancer (Table 2). This finding is very worrisome as poor knowledge of different aspects of breast cancer will greatly predispose the women to the disease and jeopardize early detection of the disease. This could be the reason for late presentation and diagnosis of breast cancer in the clinics. Most previous studies reported lack of knowledge of breast cancer among women. For instance, most of the women in Tanzania lacked adequate knowledge on breast cancer risk factors, causes, symptoms and effects^17^. More than half of females have poor knowledge regarding mammography in Rawalpindi and Islamabad City^18^. Majority of the women in Najran, Saudi Arabia demonstrated poor knowledge of breast cancer and screening methods^19^. However, another study in Al-Ahsa, Saudi Arabia reported good level of knowledge of breast cancer^20^. Our finding could be because of the general lack of basic education of the respondents. It can be seen from Table 1 that only 15.8% of the respondents had secondary education which is basic education in Nigeria. Education, monthly income and having ever been screened for breast cancer exerted significant influence on knowledge of breast cancer (Table 3). Therefore, there is a need for continued community-based breast cancer awareness and education by public health educators and community health workers on breast cancer to improve the rural women's knowledge. Improving the women's economic status can also improve their knowledge of breast cancer and screening practice. World Health Organization reported that education to increase knowledge and screening to promote early diagnosis are the two components of early detection of breast cancer^5^.

Lack of money and lack of knowledge of what breast cancer screening is all about were the most reported barriers to breast cancer screening (Table 5). This is evident from the demographic characteristics of the women as majority of the respondents earn less than 50,000 naira as monthly income and have never done any breast cancer screening. Majority of the women in Tanzania did not practice Breast Self-Examination due to lack of knowledge^17^. Lack of awareness about the facility offering mammography was reported as barriers to mammography screening in Rawalpindi and Islamabad City^18^.

Univariable analysis of factors associated with knowledge of breast cancer shows that women who have tertiary education were five times more likely to have breast cancer knowledge than those with no formal education. Those who earn 50,000 naira and above monthly were four and a half times more likely to have knowledge than those who earn less than 50,000 naira monthly while those who have ever screened were less likely to have breast cancer knowledge than those that have never screened. These finding are consistent with other studies. Educated women were more likely to have sufficient information regarding mammography screening compared to women with lower levels of education in Hungary^21^. Breast cancer screening may be determined by one's financial strength and level of education^22^. It is therefore needful to engage in advocacy visits to ensure that breast cancer screening gets as much community participation as possible. In the multivariable analysis, women that have ever screened were less likely to be willing to be screened than those never screened. This could be because the ever-screened patients perceive breast cancer development and further screening as not necessary. Quality of services at the screening center could also be a factor. Client follow-up should be given more attention by the health care personnel at the clinic to ensure that clients return to the clinics on appointment days. Again, continued health education is essential to inform women that one-off screening is not enough for early detection since the disease can develop at any time.

In conclusion, our study showed a gross lack of knowledge concerning breast cancer and its screening. Lack of knowledge and fund were the barriers preventing women from taking up the screening services. Most participants were willing to screen if screening were at no cost to them. Therefore, there is urgent need for intensified breast cancer education programme for rural women and subsidizing the cost of screening by governmental and non-governmental agencies to improve screening uptake among rural women. Moreover, empowering women economically can also improve screening uptake, as they will have the financial power to use the screening services. Future research needs to explore possibilities of funding to subsidize screening cost for the women, empowering the women economically and testing if the empowerment can lead to sustainable screening behavior.

## Strengths and limitations of the study

This study has contributed in creating breast cancer awareness among the study participants which could be leveraged on in planning future breast cancer control plans among the citizens. However, the study is bereft with some limitations. The small sample size, use of non-probability sampling technique and the use of only women found in the church limits the findings. The findings, therefore may not be generalizable to the entire population. A probability sampling method would give a more robust finding that could represent the views of entire community. Also, the use of only questionnaires has some limitations to the study. A mixed method approach will give a comprehensive view of the subject matter. However, the findings are eye opener to what the knowledge, barriers and willingness to participate in breast cancer screening among women looks like. The findings will guide the development of a comprehensive strategy to reduce breast cancer incidence which may include educational and economic empowerment programmes.
